# Echocardiographic and hemodynamic determinants of right coronary artery flow reserve and phasic flow pattern in advanced non-ischemic cardiomyopathy

**DOI:** 10.1186/1476-7120-5-31

**Published:** 2007-09-26

**Authors:** Pedro Graziosi, Barbara Ianni, Expedito Ribeiro, Marco Perin, Leonardo Beck, Claudio Meneghetti, Charles Mady, Eulogio Martinez Filho, Jose AF Ramires

**Affiliations:** 1 Heart Institute (InCor) – University of Sao Paulo Medical School, Clinical Division, Sao Paulo, Brazil

## Abstract

**Background:**

In patients with advanced non-ischemic cardiomyopathy (NIC), right-sided cardiac disturbances has prognostic implications. Right coronary artery (RCA) flow pattern and flow reserve (CFR) are not well known in this setting. The purpose of this study was to assess, in human advanced NIC, the RCA phasic flow pattern and CFR, also under right-sided cardiac disturbances, and compare with left coronary circulation. As well as to investigate any correlation between the cardiac structural, mechanical and hemodynamic parameters with RCA phasic flow pattern or CFR.

**Methods:**

Twenty four patients with dilated severe NIC were evaluated non-invasively, even by echocardiography, and also by cardiac catheterization, inclusive with Swan-Ganz catheter. Intracoronary Doppler (Flowire) data was obtained in RCA and left anterior descendent coronary artery (LAD) before and after adenosine. Resting RCA phasic pattern (diastolic/systolic) was compared between subgroups with and without pulmonary hypertension, and with and without right ventricular (RV) dysfunction; and also with LAD. RCA-CFR was compared with LAD, as well as in those subgroups. Pearson's correlation analysis was accomplished among echocardiographic (including LV fractional shortening, mass index, end systolic wall stress) more hemodynamic parameters with RCA phasic flow pattern or RCA-CFR.

**Results:**

LV fractional shortening and end diastolic diameter were 15.3 ± 3.5 % and 69.4 ± 12.2 mm. Resting RCA phasic pattern had no difference comparing subgroups with vs. without pulmonary hypertension (1.45 vs. 1.29, p = NS) either with vs. without RV dysfunction (1.47 vs. 1.23, p = NS); RCA vs. LAD was 1.35 vs. 2.85 (p < 0.001). It had no significant correlation among any cardiac mechanical or hemodynamic parameter with RCA-CFR or RCA flow pattern. RCA-CFR had no difference compared with LAD (3.38 vs. 3.34, p = NS), as well as in pulmonary hypertension (3.09 vs. 3.10, p = NS) either in RV dysfunction (3.06 vs. 3.22, p = NS) subgroups.

**Conclusion:**

In patients with chronic advanced NIC, RCA phasic flow pattern has a mild diastolic predominance, less marked than in LAD, with no effects from pulmonary artery hypertension or RV dysfunction. There is no significant correlation between any cardiac mechanical-structural or hemodynamic parameter with RCA-CFR or RCA phasic flow pattern. RCA flow reserve is still similar to LAD, independently of those right-sided cardiac disturbances.

## Background

In recent years, interest in cardiac right sided compromise in heart failure (HF) due to dilated cardiomyopathy has increased, mainly because of its prognostic implications [1,2]. The role and repercussions of right coronary artery circulation under these conditions in human being are lacking.

In HF physiopathology, several mechanical and hemodynamic disturbances can affect both right and left cardiac chambers but in different ways [3-6]. In right side, particularly pulmonary artery (or right ventricular) systolic hypertension or right ventricle (RV) dysfunction could affect the right coronary flow and pattern, as observed even in pioneering experimental studies [7-10].

The phasic coronary flow pattern, in dogs under normal intracavitary pressures, presents systolic flow predominance over diastolic flow in right coronary artery (RCA), inversely to left anterior descendent coronary artery (LAD) [7,9]. Particularly, the LAD phasic pattern is influenced by LV mechanical systolic forces [11,12]. Experimentally, when RV systolic hypertension occurs, the RCA systolic flow is attenuated because of reduction in systolic coronary driving pressure to RV [7,9]. These findings could not be extrapolated to humans, mainly due to differences with animal anatomy and physiologic conditions [13]. In normal human beings, it has been reported that diastolic flow velocity is mildly predominant over systolic flow velocity, in proximal RCA [14]. In occurrence of HF, the RV susceptibility to mechanical forces is still more expectable [3,4]. Regarding coronary flow reserve (CFR), in normal human subjects is reported an RCA/LAD equivalence [14,15]. However, in presence of dilated non-ischemic cardiomyopathy (NIC), most studies refers only to LAD [16-19], and, in someway, extrapolates to global coronary circulation, not considering the possible influences from right sided disturbances. Experimentally, under acute and marked increasing of RV systolic pressure, it was observed a decreasing in RCA flow followed by a subsequent RV failure [10].

In patients with HF resulting from chronic dilated advanced NIC, it is not known if and how RCA flow pattern and reserve are affected, even in comparison with LAD.

The purposes of this study were to evaluate the phasic flow pattern and coronary flow reserve in RCA in patients with chronic dilated non-ischemic cardiomyopathy and severe LV dysfunction, the possible influences from pulmonary arterial hypertension and RV dysfunction in this setting, and to compare these parameters to those obtained in left coronary circulation. As well as, to investigate any correlation among the cardiac structural, mechanical and hemodynamic parameters with RCA phasic flow pattern and CFR.

## Methods

### Study patients

This study included twenty-four patients with non-ischemic dilated cardiomyopathy and severe left ventricular (LV) dysfunction, who have consented to be submitted to non-invasive and invasive cardiac evaluation. The cardiovascular medications used, according to clinical indications, were basically diuretics, digitalics, angiotensin-converting enzyme inhibitors and alpha-methyldopa; no patient was in use of beta-blockers, xantine contents or anticoagulants. Concerning cardiomyopathy etiologies, we had 12 patients with hypertensive cardiomyopathy, five with chagasic, and seven with dilated idiopathic cardiomyopathy. The inclusion criteria were presence of cardiomyopathy with LV diffuse hypokinesia and severe systolic dysfunction defined by LV fractional shortening ≤ 20%, evaluated by echocardiography [20,21], in patients in follow-up at least one year in our Institution; sinus rhythm in ECG; and absence of obstructive coronary artery disease, as evaluated by coronary angiography. Exclusion criteria were: presence of blood levels of creatinin > 1,8 mg/dL and hemoglobin < 10 g/dL; stroke during previous year; advanced malignant disease; history of myocarditis; presence of significant valvular or cardiac congenital disease; and contraindications to use adenosine and to do cardiac catheterization.

The study was conducted in accordance with the Declaration of Helsinki, and the protocol was approved by the Hospital's Ethic Committee. All patients have given their written informed consent after clarification of all steps of the study.

### Study protocol

After clinical and laboratorial evaluation, the patients were submitted to 12-lead ECG, 2D-Doppler echocardiography, and RV radionuclide ventriculography. Afterwards, the patients were submitted to cardiac catheterization, coronary angiography, and intracoronary Doppler study in both RCA and LAD.

To compare parameters we also divided the patients in subgroups according to pulmonary artery systolic pressure (PASP) and according to RV function.

### Echocardiography

The echocardiograms were performed in the Hospital setting, in same period of invasive study, employing a Sequoia™ 512 (Acuson Corporation, Mountain View, California, USA) echocardiography equipment, with a broad-band transducer.

The LV dimensions, regional and global function evaluations were performed using two-dimensional and M-mode approaches accordingly to the American Society of Echocardiography [20,21] and applying established recommendations [22-24]. It was also calculated the LV fractional shortening, LV mass index and wall systolic stress [22-24]. Spectral Doppler and color flow mapping helped to exclude patients with concomitant severe valvular regurgitation. During the exams, the patients were monitored by a non-invasive blood pressure device (DX 2710, Dixtal Biomedic, Manaus, Brazil) and with an one-lead ECG on echocardiography display. The data were recorded on both video-tape and digital disk.

### Radionuclide ventriculography

Radionuclide ventriculography was performed to quantify the RV ejection fraction (EF) [25,26]. The ventricular images were obtained in-hospital in a scintillation camera (LEM+, Siemens, Munchen, Germany) with a LEAP colimator, with ECG synchronization. Patients' red blood cells were labeled with 20 to 30 mCi of technetium-99 m using the modified *in vivo *technique. Data were acquired in an ECG-synchronized frame mode (32 frames/cycle with 150,000 to 200,000 counts/frames) in a 64 × 64 computer matrix. Multiple-gated equilibrium blood pool imaging was performed at rest to determine global RV EF.

According to RV EF values we separated two groups for data comparison, one considered with effective RV dysfunction, RV EF < 0.35 (12 patients), and other named with preserved function, RV EF > 0.35 (12 patients).

### Cardiac catheterization

Just before the coronary angiography, right and left cardiac catheterization were done, according Judkins technique, and also it was obtained measurements by a Swan-Ganz catheter (139F75 model, Baxter Healthcare Corporation, Irvine, California, USA) with support of a data register (EP2 DT-CAT 2 model, BESE – Bio Engenharia de Sistemas e Equipamentos S.A., Belo Horizonte, MG, Brazil). A cardiac catheterization equipment H-3000 (Philips Medical Systems Ltda, Eidhoven, Netherlands) was employed.

The cardiovascular medications were suspended 24 hours before the study according to the clinical individual condition. The patients were advised to not use xantines, caffeine, or cola contents, in the preceding 24 hours.

The PASP was assumed to be the same as the systolic RV pressure once in the absence of ventricular obstruction [27]. A PASP equal or above 35 mm Hg was considered as pulmonary hypertension [27]. Thus, we could separate two groups for data comparison: without (< 35 mm Hg; 14 patients) and with pulmonary arterial hypertension (≥ 35 mm Hg; 10 patients).

### Coronary angiography

All patients underwent selective coronary angiography using a 7F standard catheters and proceeding conventional views. On completion of diagnostic cardiac catheterization, the digital record of the procedure was reviewed. Only patients whose coronary arteries were angiographically normal were enrolled in the study. It was used a quantitative angiographic digital analysis system (CAAS II, Pie Medical, Maastricht, Netherlands) to analyze coronary lumen [28]. Right coronary circulation dominance was defined applying established criteria [29].

### Coronary-flow velocity measurements

Consecutively to coronary angiography, the left and right coronary arteries (in random order) were selectively engaged with a diagnostic catheter. All patients received 7500 IU bolus of heparin, and also an intracoronary 5 mg isosorbide-5-mononitrate bolus was done before the procedure to prevent catheter-induced coronary artery spasm and to minimize changes in coronary artery diameter [30]. The Doppler signal were obtained after an interval for wash-out from the contrast agent infusion. A 0.014-in., 15-MHz Doppler guide wire (FloWire^®^, EndoSonics, Inc., Rancho Cordova, California, USA) was advanced through the catheter to the proximal LAD, away at least 10 mm from a septal branch, and then to proximal RCA. Baseline flow-velocity measurements were performed once a stable Doppler signal was obtained. Frequency analysis of the Doppler signals was carried out in real time by fast Fourier transform using a velocimeter (FloMap^®^, EndoSonics, Inc., Rancho Cordova, California, USA). Over the Doppler envelope was calculated the time-average peak flow velocity (APV) obtained from the last two cardiac cycles as the image was frozen. The Doppler velocity signals were displayed along with simultaneous ECG and aortic pressure waveforms, and the envelopes were automatically analyzed using the FloMap^® ^equipment system. All registers were printed and recorded on videotape. Once baseline flow-velocity data had been obtained, we have used the best signal resulting from two ways to obtain the maximal hyperemic signal: i.e., a bolus injection of intracoronary adenosine (Adenocard^®^, Libbs Farmaceutics, Brazil), 18 μg for LAD and 12 μg for RCA, and after a wash-out step, an intravenous adenosine continuous infusion (140 μg/kg/min) [31,32], employing a high precision infusion pump (ANNE^®^, Anesthesia Infusion System, Abbott Laboratories, Illinois, USA) to LAD and RCA evaluations, respectively. The CFR equivalence from these two acquisitions ways is already established [31,32]. The CFR was defined as a function of the highest value obtained from any manner. CFR was determined as the ratio of the time-averaged peak coronary flow velocity after adenosine administration to the time-averaged peak coronary flow velocity at baseline, for both left and right coronary arteries. We assumed that coronary flow velocity reserve was representative of CFR [33]. The phasic coronary flow pattern was defined as diastolic/systolic ratio from the corresponding time-averaged peak coronary flow velocity signal.

### Statistical analysis

Data were expressed as mean values ± SD. Non-parametric Mann-Whitney test was used to compare the mean values of two subgroups (independent samples). Non-parametric Wilcoxon test was used to compare two conditions in the same group (related samples). The data were also represented in box-plots expressed as median ± SE. The Pearson's correlation was employed for the measurement of association between the different analyzed variables. A probability value of less than 0.05 was considered as statistically significant. A computer analysis system SPSS software version 8.0 (SPSS, Chicago, Illinois, USA) was employed to support the statistical analysis.

## Results

### Patients characteristics

We enrolled twenty-four patients with dilated non-ischemic cardiomyopathies and severe LV dysfunction, 15 men, with mean age 50.7 ± 10 years, all with symptoms more than one year and heart failure functional class (NYHA) II or III, by occasion of the evaluation. The LV echocardiography variables are shown in Table 1. We could observe, in average, a severe LV dysfunction and an important increase in LV diastolic diameter, likewise in LV mass index and end systolic wall stress, in accordance with the advanced LV mechanical and structural damage. The RV function, analyzed by radionuclide ventriculography, have presented a RV EF mean value of 0.35 ± 0.11 for the entire group of patients, and it was possible to divide two subgroups to comparison according to ejection fraction. We met a significant RV EF difference between the named preserved and non-preserved RV function subgroups (respectively, 0.44 ± 0.06 vs. 0.26 ± 0.08, p < 0.01).

**Table 1:** Left ventricle structural characteristics and hemodynamic data

	**Mean**	**SD**
Fractional shortening (%)	15.30	3.50
End diastolic diameter (mm)	69.40	12.20
End systolic diameter (mm)	59.00	10.30
Mass index (g/m^2^)	232.60	68.70
Volume/mass (mL/g)	0.88	0.33
End systolic wall stress (10^3^·dyn/cm^2^)	158.30	50.60
Aorta systolic pressure (mm Hg)	114.00	4.63
Aorta diastolic pressure (mm Hg)	69.70	2.11
Pulmonary artery systolic pressure (mm Hg)	34.70	3.16
Right atrium mean pressure (mm Hg)	7.80	0.88
Pulmonary vascular resistance (Wood units)	1.71	0.18
Pulmonary capillary pressure (mm Hg)	15.00	1.90

Table 1 shows also the hemodynamic profile obtained invasively. From that we divided two subgroups according to pulmonary arterial systolic pressure, with a significant difference between the normal vs. pulmonary arterial hypertension subgroups values (respectively, 23.80 ± 4.76 vs. 50.10 ± 11.45 mm Hg, p < 0.01).

The right coronary circulation dominance was present in 96% of patients. No patients had coronary obstructions. We had no major complications in any invasive procedure.

### Phasic coronary flow pattern

**Figure 1:**
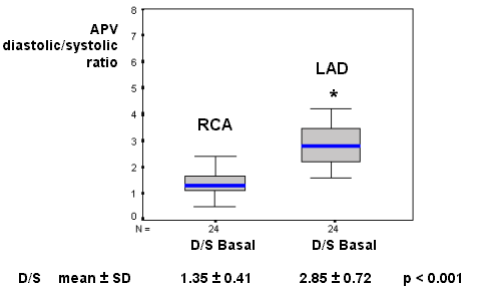
Box-plot representing the RCA vs LAD comparison regarding the phasic coronary flow pattern (D/S), showing a diastolic mild predominance in RCA, that is significantly more marked in LAD. APV – time-averaged peak coronary flow velocity; Basal – resting condition; D/S – diastolic/systolic APV ratio; N – number of patients; LAD – left anterior descending coronary artery; RCA – right coronary artery.

**Figure 2:**
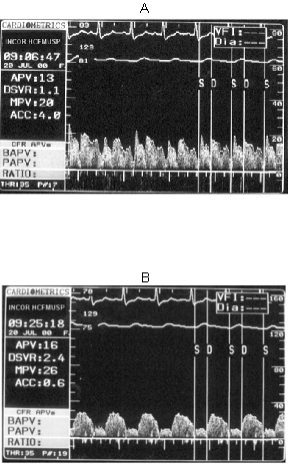
Baseline spectral Doppler coronary flow velocity signal in right coronary artery (A) and left anterior descending coronary artery (B). S = systolic, D = diastolic, portions of phasic coronary flow. APV = time-averaged peak coronary flow velocity. DSVR = diastolic/systolic flow velocity ratio.

**Figure 3:**
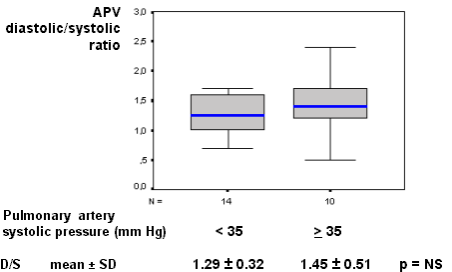
Box-plot representing the RCA phasic coronary flow pattern (D/S) according the pulmonary artery systolic pressure, showing no difference between pulmonary non-hypertensive and hypertensive subgroups. APV – time-averaged peak coronary flow velocity; D/S – diastolic/systolic APV ratio; N – number of patients; RCA – right coronary artery.

**Figure 4:**
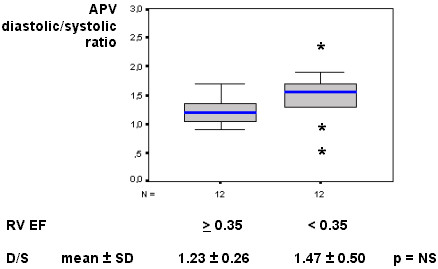
Box-plot representing the RCA phasic coronary flow pattern (D/S) according the RV ejection fraction, showing no difference between RV non-dysfunctional vs. dysfunctional subgroups. APV – time-averaged peak coronary flow velocity; D/S – diastolic/systolic APV ratio; N – number of patients; RCA – right coronary artery; RV EF – right ventricular ejection fraction.

Our results showed a resting mild diastolic predominance on RCA phasic flow pattern (diastolic/systolic APV ratio = 1.35); this RCA diastolic/systolic phasic flow pattern was significantly lesser than in LAD (respectively, 1.35 vs. 2.85, p < 0.001), as represented in Figure 1 and exemplified in Figure 2. The RCA phasic flow pattern was not different between subgroups with and without pulmonary arterial hypertension (respectively, 1.45 vs. 1.29, p = NS), as demonstrated in Figure 3; and even comparing subgroups with and without RV dysfunction (respectively, 1.47 vs. 1.23, p = NS), as showed in Figure 4. We have no found significant correlation between the hemodynamic and echocardiographic parameters and the RCA phasic flow pattern, as described in Table 2.

**Table 2:** Echocardiographic and hemodynamics parameters correlation with right coronary phasic flow pattern

	Pearson correlation coefficient	p
LV Fractional shortening (%)	0,14	0,52
LV mass (g)	-0,06	0,78
LV mass index (g/m2)	-0,11	0,62
End systolic wall stress (10^3^·dyn/cm2)	-0,32	0,13
LV volume/mass ratio (mL/g)	0,43	-0,17
RV ejection fraction	-0,07	0,73
Cardiac index (L/min/m2)	-0,03	0,88
Pulmonary artery systolic pressure (mm Hg)	0,29	0,18
Pulmonary capillary pressure (mm Hg)	0,28	0,19
Pulmonary vascular resistance (Wood unit)	0,16	0,46
Systolic pressure gradient between aorta-RV (mm Hg)	-0,25	0,23
Gradient between diastolic aortic and mean right atrial pressure (mm Hg)	-0,16	0,44

p = descriptive level of Pearson correlation

### Coronary flow reserve

**Figure 5:**
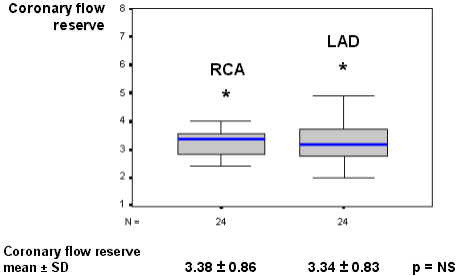
Box-plot representing the RCA vs LAD comparison respecting the coronary flow reserve, showing no significant difference. LAD – left anterior descending coronary artery; N – number of patients; RCA – right coronary artery.

**Figure 6:**
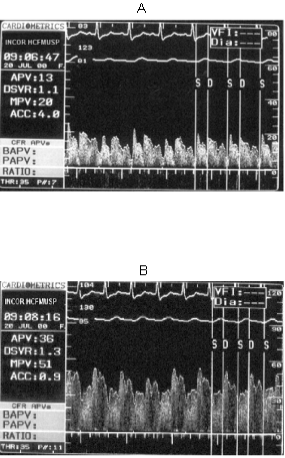
Right coronary artery spectral Doppler coronary flow velocity signal in baseline (A) and hyperemic (B) conditions. S = systolic, D = diastolic, portions of phasic coronary flow. APV = time-averaged peak coronary flow velocity. DSVR = diastolic/systolic flow velocity ratio.

RCA flow reserve was not different in comparison to LAD (respectively, 3.38 vs. 3.34, p = NS), as showed in Figure 5. The heart rate ranged from 80.2 ± 11.8 to 83.5 ± 13.0 beats/min (p = NS), and mean arterial blood pressure ranged from 79.4 ± 14.9 to 74.6 ± 14.8 mm Hg (p = NS), from basal to hyperemic condition, respectively. RCA resting flow presented a significant increasing after adenosine hyperemic stimulus (11.6 vs. 38.6, p < 0.001, in cm/s). The RCA flow velocity hyperemic increment is exemplified in Figure 6. There was no difference comparing RCA-CFR to LAD-CFR in the pulmonary hypertension subgroup, as well as in the RV dysfunctional subgroup, as exposed in Table 3. We have no met significant correlation between the hemodynamic and echocardiographic parameters and the RCA flow reserve, as described in Table 4.

**Table 3:** RCA vs LAD CFR in pulmonary hypertension and RV dysfunction subgroups

	**RCA**	**LAD**	**p**
PSAP ≥ 35 (50 ± 11) mm Hg mean CFR	3.09 ± 0.48	3.10 ± 0.62	NS
RV EF < 0. 35 (0.26 ± 0.06) mean CFR	3.06 ± 0.47	3.22 ± 0.57	NS

It had no difference comparing RCA vs. LAD CFR in pulmonary hypertension and RV dysfunction subgroups.

CFR – coronary flow velocity reserve

RCA – right coronary artery

LAD – left anterior descending coronary artery

PASP – pulmonary artery systolic pressure

RV – right ventricle/ventricular

EF – ejection fraction

**Table 4:** Echocardiographic and hemodynamics parameters correlation with right coronary flow reserve

	Pearson correlation coefficient	p
LV Fractional shortening (%)	0,28	0,18
LV mass (g)	-0,19	0,37
LV mass index (g/m^2^)	-0,07	0,76
End systolic wall stress (10^3^·dyn/cm^2^)	-0,28	0,19
LV volume/mass ratio (mL/g)	-0,18	0,40
RV ejection fraction	0,40	0,06
Cardiac index (L/min/m^2^)	0,06	0,79
Pulmonary artery systolic pressure (mm Hg)	-0,26	0,22
Pulmonary capillary pressure (mm Hg)	0,30	0,15
Pulmonary vascular resistance (Wood unit)	-0,10	0,63
Systolic pressure gradient between aorta-RV (mm Hg)	0,33	0,11
Gradient between diastolic aortic and mean right atrial pressure (mm Hg)	0,42	0,04

p = descriptive level of Pearson correlation

## Discussion

This study is first evaluating RCA flow velocity pattern and flow reserve in patients with HF resulting from advanced non-ischemic cardiomyopathy, employing invasive spectral Doppler technique. We showed that in these patients RCA phasic flow pattern has still a mild diastolic predominance, contrarily from verified experimentally [7-9], and that was not significantly influenced by occurrence of pulmonary hypertension or RV dysfunction (Figs. 1, 2, 3, 4). Aside, we have no observed any significant correlation with echocardiographic and hemodynamic parameters with RCA flow pattern or RCA-CFR (Table 1). Moreover, the RCA flow reserve did not differ from LAD, even when pulmonary hypertension or RV dysfunction were present (Fig 5, Table 3).

In fact, most of reported studies involving RCA phasic flow or CFR in this HF setting is experimental. In human with non-obstructive coronary disease, the vast majority of investigations refers to patients with others cardiopathies or even to normal subjects, or employing another techniques, or just focusing only LAD [16-19], limiting therefore the pertinent reasoning.

### Coronary phasic flow pattern

Our findings showed a mild diastolic predominance in RCA flow pattern (Figs. 1 and 2), differently from some animal experiments also in face of hemodynamic abnormalities, in which it was observed a systolic flow preponderance [7-9]. Actually, in dogs RCA is small and limited to RV [13], so its branches do not receive influence from vigorous LV contraction over intramural resistance vessels during systole, which is considered a main responsible in attenuating systolic flow [11].

In our study, the LAD diastolic flow predominance was marked, compatible with expected LV mechanics influence (Figs. 1 and 2). In fact, Krams et al. have shown experimentally that the LV contractility or elastance were most responsible to restricting systolic coronary flow, even compared to intracavitary pressure [12,34]. Also in normal human being it was observed a systolic flow attenuation in LAD [14]. Akasaka et al. studying patients with hypertrophic cardiomiopathy [35], as well as Yoshikawa et al. studying patients with aortic stenosis [36], have shown a still more distinct impediment in systolic component, or even a reverse systolic flow.

RCA dominance is present in majority of humans [29], as it was observed in our patients, so once RCA branches reach and penetrate LV myocardium, we can assume that LV muscle contraction should also influence in any degree the RCA phasic flow pattern, explaining part of our findings. Possibly, in a small RCA marginal branch, just restrict to RV and not representative of entire RCA ramifications, and particularly in a more distal coronary analysis, we could find a diverse coronary flow pattern, as observed by Okura et al. [37] and others investigators [38,39] studying another cardiopathies and circumstances.

Right cardiac side has several structural and functional differences from left cardiac side [3-6]. For example, RV free wall is generally thinner than LV wall, in normal and in patients with dilated cardiomyopathy [4,6]. Therefore, we could infer that also in human being, the RV intraventricular pressure, or even the RV wall stress, could have still more influence in the mechanics of coronary flow pattern [3,6]. Nevertheless, also in patients with pulmonary hypertension or with RV structural and functional repercussions, we have not verified any effect in RCA flow pattern. Although such influence have ever been demonstrated in animals – as in Lowensohn's study [9], particularly analyzing an extremely increased RV intracavitary pressure – these acute and non-physiological experimental conditions could not represent the chronic compensated human HF state, even under pulmonary hypertension condition. The significant RV dysfunction – which represent the final common pathway of diverse structural and mechanical RV abnormalities [3,4] – have not influenced significantly the RCA flow pattern, in comparison to preserved RV functional subgroup (Fig. 4). Other than, the complex RV structure may have influence from LV disarrangement [3,4,6], aside from the dysfunctional interventricular septum. It is inferably that in this chronic and stable advanced cardiomyopathy HF setting, these disturbances in right cardiac side were not robust enough to exacerbate RCA systolic flow attenuation. For those same reasons we attributed the absence of significant correlation among any isolated echocardiographic and hemodynamic parameters and RCA phasic flow pattern, as described on Table 2. So, the RCA balanced contact with both RV and LV, and their respective simultaneous structural and hemodynamic contributions, probably is one of the main reasons for this lacking of any robust correlation.

### Coronary flow reserve

Independently of inherent mechanical and hemodynamic differences between right and left cardiac sides in advanced heart failure due to dilated non-ischemic cardiomyopathy [2-4,6,40], the CFR was equivalent in RCA and left coronary circulation (Fig. 5). In settings without such deterioration, i.e. in normal human beings, Ofilli et al. have reported a balanced RCA versus LAD flow reserve, employing alike technique [14]. The several vascular intrinsic and extrinsic mechanisms enrolled in preserving the CFR (19) possibly were in account to promote our findings. Inclusive, the former mechanism has surely a diffuse nature in the cardiac vessels, as already it was verified experimentally [41].

We have employed adenosine, to provoke hyperemia, that is fundamentally an endothelium-independent stimulant and acts at arteriolar level [31]. Thus, instead of analyze intrinsic factors, we have assessed mostly the resistance vessels, that are particularly under mechanical extrinsical influences [19,42]. For sure, LV structural disturbances – that include since wall and cavity compromising until perivascular disarrangement – had any kind of effect on CFR [19]. Additionally to any RV compromising level, certainly the RCA extension to LV, prominent in RCA dominance circumstances, had an important role to maintaining that CFR equivalence. Probably, these are also the explanations for the lacking of significant correlation of RCA-CFR with any echocardiographic or hemodynamic variable isolatedly, once their effects were consequently minimized (Table 4). These findings are contrastable to the assessment of LAD-CFR and the correlation with LV parameters exclusively, as verified by Inoue et al. [16].

The presence of pulmonary arterial hypertension has also a prognostic role in advanced HF patients [27]. When we have compared the RCA to LAD flow reserve in the pulmonary hypertension patients group, we did not find a significant difference (Table 3). Fixler et al, have found a decrease in coronary flow, and a subsequent RV failure, in setting of markedly high RV intraventricular pressure, in dogs [10]. As a matter of fact, at initials levels of RV hypertension, the RCA flow even increases, corresponding to a vasomotor adaptation and reserve, including in acute conditions [10,43]. Only when a more extreme RV systolic pressure is reached, this vasomotor reserve exhausts, then occurring that hemodynamic consequences [10]. Nevertheless, Manohar et al. had observed experimentally in ponies that even in presence of an elevated right intraventricular pressure, and a consequent marked driving pressure reduction, the RCA flow reserve was preserved [13]. This RCA-CFR capability to overcome hemodynamic adversities was observed experimentally by Murakami et al. as well, employing also adenosine in dogs [44]. Certainly, this evidenced capability range of CFR adaptation – added to RCA interaction to both RV and LV, as observed in humans and also in ponies [13] – and the, not experimental, chronic and relatively clinical stable conditions found in our patients, have contributed to our balanced findings. Other than, the not significant heart rate variation during the stress, in our patients, also could have a role in RCA-CFR preservation, as remarked in Manohar's study [13]. In RV dysfunctional circumstances, we also did not find a difference between RCA and LAD flow reserve (Table 3). The RV functional assessment in this HF setting was also appealing due to particular prognostic implications [1,2,4]. However, the presence of significant RV dysfunction have not influenced the RCA-CFR enough to make a distinction with the left arterial CFR. As a matter of fact, there are many different reasons influencing RV systolic function [3-5], particularly in advanced NIC scenario, making any correlation with RV function difficult. Some of such reasons could be the LV-RV muscle contiguity, ventricular interdependence, direct injuries to RV myocardial fibers, or even the high pulmonary artery pressure *per se *[3,4]. Apart from that, the ejection fraction, as an ejection phase index, is influenced by load conditions, hence limiting the effective myocardium fiber contraction analysis [3-5]. It is possible also, that a presence of any kind of vasomotion corresponding interaction in LAD as RCA be stressed [43]. Therefore, aside from the different mechanisms in preserving CFR, it is reasonable to accept the weak relationship found in this study enrolling RV dysfunction over RCA versus LAD CFR, in conditions of advanced chronic non-ischemic heart failure syndrome.

Although it has not been object of the present study, the absolute increment verified on CFR merits some considerations. Firstly, we had no control group to compare with and thus to endorse these findings. Secondly, even though we had analyzed advanced non-ischemic cardiomyopathies, the different etiologies could have a role in our findings. In fact, few studies have evaluated CFR in Chagas' disease. Torres et al. have found a marked reduction of CFR with acetylcholine, not paralleled with the adenosine infusion [18]. We have also observed a preserved adenosine CFR in another study, but assessing Chagas' heart disease with no LV dysfunction [45]. Some older studies assessing CRF in dilated cardiomyopathies have found a diverse CFR impairment comparing endothelium with non-endothelium stimulation [17,19]. Treasure et al. analyzing CFR in dilated cardiomyopathy have found a significant reduction under acetylcholine stimulus, but it had no difference comparing with controls under adenosine provocation [17]. By other hand, Bitar et al have reported a variable response on coronary arteries after acetylcholine infusion in non-ischemic dilated cardiomyopathy HF setting [46]. Vanderheyden et al. have verified a blunted adenosine CFR in idiopathic dilated cardiomyopathy, but they speculated the higher resting coronary flow as one of the reasons for these findings [47]. Nevertheless, most of recent studies accords with the non endothelium-dependent (i.e., adenosine and dipyridamole) CFR reduction in dilated non-ischemic cardiomyopathy setting, principally studying LAD territory – justified mainly due LV structural and mechanics deterioration aside the microvascular disarrangements, as described by Rigo et al. and others [16,19,47,48]. Thirdly, it is worth of note that we have used a more incisive adenosine protocol to looking for the highest hyperemic peak. Fourthly, it is known that the CFR in this HF setting could present individually some diversity, maybe being lower in most advanced cases, or even more related with HF functional class, as reported by Santagata et al. [49]. And finally, as commented by Nitenberg and Antony, it has a lot of variables influencing the CFR assessment, considering the different techniques, protocols and studied population, therefore demanding meticulousness in comparative analysis from different studies [50].

### Clinical implications

This study demonstrated how is the RCA flow behavior in conditions of HF in advanced non-ischemic dilated cardiomyopathy. Although the RCA phasic flow pattern had been different from that evidenced in experimental studies [7,9], we met similar findings than described in normal human being [14,15]. Certainly, these findings are in accordance with the several mechanisms of coronary flow adaptation in human being [19]. Other than, it is possible to suppose that any process involved in the diastolic function improvement also could ameliorate the predominant diastolic coronary flow, in not only the left but also in the right cardiac side, in this HF setting [51,52]. Hence, minimizing the compromised systolic component, and then creating a favorable vicious circle, inclusive with a benign repercussion on ventricular systolic function. These aspects could have even therapeutic implications. Also, it is noteworthy that it is still technically difficult to get some information non-invasively from RCA [53], mainly detailed spectral Doppler curves to diastolic/systolic proportion analyses, making primary, at the moment, to employ invasive studies to improve these human RCA physiopathology understanding.

Respecting CFR, by these results we can infer that is possible to extrapolate the findings from LAD to RCA, or vice versa, in this setting of patients. Generally it is easier to explore the CFR in left coronary circulation (LAD) due to its topography [16-19], even non-invasively [48,53,54], avoiding time-consuming and the technical limitations concerning RCA circulation assessment. This information has more importance when one wishes to evaluate the global non-regional microcirculation, in a given non-ischemic cardiomyopathy population, employing invasive or non-invasive Doppler techniques [18,19,48,53,54]. Rigo et al have recently demonstrated the prognostic importance of CFR, employing transthoracic dipyridamole echocardiography at left coronary artery territory in patients with dilated NIC [48]. Such study have permitted inclusive to recommend particular treatment strategies based on CFR behavior [48]. Our study could endorse the diffuse nature of CFR findings at chronic dilated NIC setting, relatively independent of right cardiac repercussions. Apart this, to assert that efforts to improve RCA-CFR could have a role in benefiting RV failure in advanced NIC or even in survival, it will require more investigations.

### Study limitations

This study had some limitations. Regarding the different cardiomyopathy etiologies, we have considered that as the aim was determine fundamentally the cardiac mechanical and hemodynamic repercussions in coronary flow dynamics, we have emphasized mostly the advanced structural stage from chronic non-ischemic cardiomyopathies, independently of its etiology. Normal subjects were not evaluated due to ethical issues. Another limitation is the number of patients; however, the invasive nature of studies, additionally its high costs, and sufficient statistical basis, made this number of patients enough. Concerning the two methods of hyperemia induction, which are equivalents according other pertinent studies [31,32], we have decided to employ both in order to avoid technical limitations, thus reducing case exclusions, and permitting acquisition of a more accurate and stable hyperemic Doppler signal. The acquisition of Doppler flow velocity signal in proximal coronary segment could present any different behavior comparing to the distal signal [38]. We avoid this latter approach because, besides to normalization reasons, we considered that distal acquisition could not represent the global RCA tree, and also could imply higher coronary manipulation risks.

## Conclusion

In patients with chronic non-ischemic dilated cardiomyopathy and severe LV dysfunction, the RCA phasic flow pattern has yet a mild diastolic predominance, less marked than LAD, and it is not significantly influenced by presence of pulmonary hypertension or RV dysfunction. Neither RCA phasic flow pattern nor RCA-CFR presented significant correlation with any cardiac structural, mechanical or hemodynamic parameter. RCA flow reserve is similar to LAD in these patients, even in presence of pulmonary arterial hypertension or RV dysfunction. In this setting of patients, it could be reasonable to extend to RCA the flow reserve parameters acquired from LAD, where they are more easily obtainable even non-invasively with echocardiography.

## Abbreviations

APV – time-averaged peak flow velocity

CFR – coronary flow velocity reserve

EF – ejection fraction

HF – heart failure

LAD – left anterior descending coronary artery

LV – left ventricle/ventricular

PASP – pulmonary artery systolic pressure

RCA – right coronary artery

RV – right ventricle/ventricular

## Competing interests

The author(s) declare that they have no competing interests.

## Authors' contributions

PG participated of study's conception and design, performance of echocardiograms, clinical and scientific support during intracoronary Doppler and hemodynamic exams; data interpretation and wrote the manuscript. BI participated in the clinical support, data acquisition, and critically manuscript revising. ER, MP and LB participated in hemodynamic exam performance and giving important suggestions. CMe coordinated the nuclear medicine exams. CMa participated in clinical support coordination and gave important contributions. EMF participated in hemodynamic exam performance and gave important suggestions. JAFR participated of study's conception and design, data interpretation, and critically manuscript revising for important intellectual content. All authors have read and approved the final manuscript.
